# Vasculature-On-A-Chip for In Vitro Disease Models

**DOI:** 10.3390/bioengineering4010008

**Published:** 2017-01-24

**Authors:** Seunggyu Kim, Wanho Kim, Seongjin Lim, Jessie S. Jeon

**Affiliations:** Department of Mechanical Engineering, Korea Advanced Institute of Science and Technology, 291 Daehak-ro, Yuseong-gu, Daejeon 34141, Korea; ksg5825@kaist.ac.kr (S.K.); do3ob@kaist.ac.kr (W.K.); iamsj@kaist.ac.kr (S.L.)

**Keywords:** vasculature-on-a-chip, angiogenesis, microfluidics, in vitro disease models

## Abstract

Vascularization, the formation of new blood vessels, is an essential biological process. As the vasculature is involved in various fundamental physiological phenomena and closely related to several human diseases, it is imperative that substantial research is conducted on characterizing the vasculature and its related diseases. A significant evolution has been made to describe the vascularization process so that in vitro recapitulation of vascularization is possible. The current microfluidic systems allow elaborative research on the effects of various cues for vascularization, and furthermore, in vitro technologies have a great potential for being applied to the vascular disease models for studying pathological events and developing drug screening platforms. Here, we review methods of fabrication for microfluidic assays and inducing factors for vascularization. We also discuss applications using engineered vasculature such as in vitro vascular disease models, vasculature in organ-on-chips and drug screening platforms.

## 1. Introduction

The vascular system, being in contact with various types of tissues and organs, is found throughout the body, and is also the basis of life mechanisms [1,2]. The functional abnormalities of the vascular system are closely related to some critical diseases such as tumor angiogenesis and cancer metastasis as well as diseases of the blood vessels themselves [3,4,5]. Thus, there have been long-standing interests in the study of blood vessels and related diseases using experimental animal models and in vitro two-dimensional static experimental platforms. However, the results from animal experiments are neither always applicable to humans, nor free from ethical concerns [6]. On the other hand, common static platforms have difficulty in clarifying some of the essential mechanisms related to human vascular diseases due to dimensional differences and the absence of the physiological level of fluid flow [7]. Meanwhile, as the advantages of the in vitro engineered vasculature emerge, enormous efforts have been placed on developing and optimizing the vascular system in vitro. Notably, in the last decade, the development of in vitro vascular systems has been accelerated by advances in microfluidic platforms [8,9,10,11]. Advantages of microfluidic chips include the ability to control the flow in physiological levels [12,13], making a 3-D microenvironment by using hydrogel as a scaffold [14,15,16], mimicking relevant tissues by incorporating human cells [17,18,19], and controlling the distribution of chemical variables [20,21]. Therefore, microfluidic devices can be used not only as a means to elucidate the mechanisms in the development of vasculatures, but also as experimental platforms for vascular disease models and drug screening [22,23,24,25].

In this review, the various types of microfluidic-based systems for generating the vasculature are introduced and discussed. Next, different factors considered in inducing vascularization on microfluidic devices are highlighted. Finally, among numerous applications using engineered vasculature, representative studies such as in vitro vascular disease models, vasculature-incorporated organ-on-chips, and drug screening platforms are investigated. This review will help readers gain a deeper understanding of vessel generations and their utilization for studying related diseases in microfluidic systems.

## 2. Vascularization in Microfluidic-Based Platforms

Until now, microfluidic systems have led advances in technology to reconstruct vessels in vitro, and many types of research have contributed to their development. Moreover, the integrations of microfluidics into the study of vascularization are in diverse forms. In this section, the typical methods used in the previous studies have been categorized into four groups: cell patterning, sacrificial molds, patterned microchannel, and self-assembly.

### 2.1. Cell Patterning

**Figure 1:**
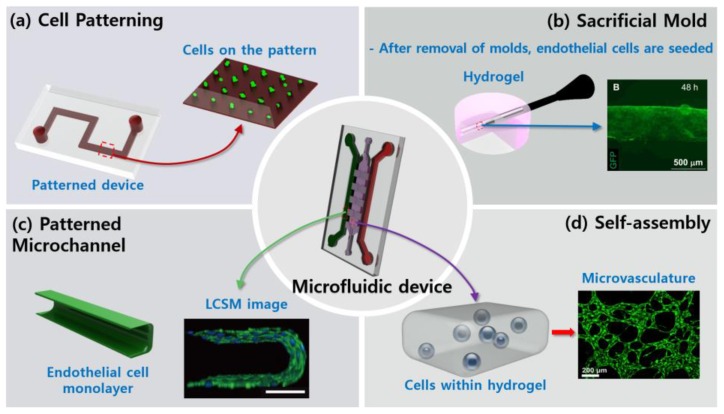
Schematic representation of fabrication methods. (**a**) Endothelial cells (ECs) (green) are cultured on the designed pattern or the specified membrane. (**b**) After removal of sacrificial molds, ECs (green) are seeded [30]. (**c**) ECs form monolayer alongside the hydrogel. Immunostaining of vascular endothelial cadherin (VE-cadherin, green) and nuclei (blue) confirm the functionality of the vessel [16]. Scale bar 100 µm. (**d**) ECs (green) are seeded within the hydrogel, and microvasculature is then formed through vasculogenesis [23]. Reproduced with permission.

The cell patterning method is achieved by marking a particular area in the device and plating cells on the marked pattern (Figure 1a). This method is a high-throughput process and is advantageous as the method is only moderately complicated to apply and control flow in the device. A method developed by Wang et al. used photolithography to create a pattern on the assay, and human umbilical vein endothelial cells (HUVECs) were later cultured on the same pattern [26]. Using a similar method with membranes, Raasch et al., made a biochip which was perfusable two-dimensionally [27]. Similarly, Young et al., placed endothelial cells (ECs) on the membrane, and measured permeability under flow [28]. Xu et al., stacked three polydimethylsiloxane (PDMS) layers and two PDMS porous membrane layers to mimic metastasis of lung cancer cells [29].

### 2.2. Sacrificial Molds

The second method for vascularization in microfluidics aims to secure spaces with structures, and the temporary molds are removed just before the cell seeding (Figure 1b). Baker et al., used gelatin as the sacrificial construct within a collagen gel [9]. After dissolving the gelatin, HUVECs were seeded, which later formed a vasculature along the collagen gel. Instead of sacrificial molds, needles or rods can also be used in microfluidic devices [20,31,32]. Nguyen et al., observed angiogenic sprouting after extraction of a needle [20], and Buchanan et al., investigated the correlation between shear stress and the effects of tumor-expressed angiogenic factors [33]. Furthermore, the effects of needle size, gelling temperature, and collagen concentration were also identified [34].

### 2.3. Patterned Microchannel

The patterned microchannel method is a process, which makes patterns using soft lithography in a device, and fills a hydrogel and cells into the empty channels (Figure 1c). Using this method, the designed channels determine the size and direction of the newly-formed vessels. This widespread method is advantageous in that it is a high-throughput process and the mechanical and biochemical stimuli are easy to control. To test the effects of drugs on vascular structures, for example, Bischel et al. [35] and van der Meer et al. [36] fabricated a 3-D lumen structure with a circular cross-section within the extra-cellular matrix (ECM). A study by Wang et al. [37] demonstrated that a cellulose-based tube could work as a patterned microchannel. They implanted a circular tube in the collagen gel and an engineered blood vessel was formed alongside the tube. The microfluidic assay of Lee et al. reproduced tumor angiogenesis and intravasation of circulating tumor cells (CTCs) by co-culturing HUVECs, lung fibroblasts, and cancer cells [38]. Zervantonakis et al. analyzed interactions between HUVCEs, cancer cells, and macrophages [39]. Wood et al., controlled diameters of vasculatures with matrix metalloproteinases (MMPs) inhibition and fluid flow [40]. In another approach, Jusoh et al. added hydroxyapatite to the fibrin gel, and generated 3-D vascular networks to mimic bone microenvironments [41]. Additionally, implantable angiogenic systems that are made of poly lactic-co-glycolic acid (PLGA) and human endothelial progenitor cells (hEPCs) were developed in vitro and later transplanted into in vivo mice [42,43].

### 2.4. Self-Assembly

The self-assembly microfluidic approach for vasculature is in stark contrast to the other methods. This approach does not require an extra structure to guide vasculatures; vascular cells are seeded within hydrogels in the device, where the cells reconstitute 3-D vascular networks by themselves so that the vasculature generated is more physiological than the ones in other methods (Figure 1d). For instance, Song et al., reproduced anastomosis using fluid control [13]. Likewise, Wang et al., by engineering artificial vascular networks, replicated anastomosis from the artery to vein [44]. This model consisted of a human endothelial colony forming cell-derived ECs and normal human lung fibroblasts in fibrin gel. Kim et al., investigated the effects of flow and pro-angiogenic factors on the growth of the vasculature, and identified the significant roles of interstitial flow [45]. Jeon et al., cultured bone marrow-derived human mesenchymal stem cells (BM-hMSCs) and HUVECs together in the microfluidic device to make functional microvasculature [19]. The same research group also formed microvasculature in the bone or muscle-mimicking microenvironments to study organ-specific metastasis [23].

## 3. Inducing Factors of Vascularization on a Chip

Vessels are mainly formed through two generic processes in vivo: vasculogenesis and angiogenesis. In vasculogenesis, endothelial precursor cells that have not yet formed lumen are differentiated to form new blood vessels [46], and angiogenesis is a process where existing blood vessels form an extensive vascular network [47,48]. There are three primary inducing factors in the formation of vasculatures: mechanical, chemical, and biological factors [11,49]. The following examples are vasculature-on-chips with the factors considered.

### 3.1. Mechanical Factors

Forces from fluid flow strongly influence ECs [50]. First of all, ECs in vessels are exposed to shear force which is tangential to the endothelial surface [12]. Shear stress causes ECs to secrete biomolecular signals [49], leading to vascular structure remodeling [46], cytoskeleton rearrangement [51,52,53], and transcriptional gene expression [47]. Furthermore, ECs are exposed to interstitial flow which acts as a transverse force across the vessel wall [12]. Interstitial flow above a certain threshold promotes capillary formation [54,55].

**Figure 2:**
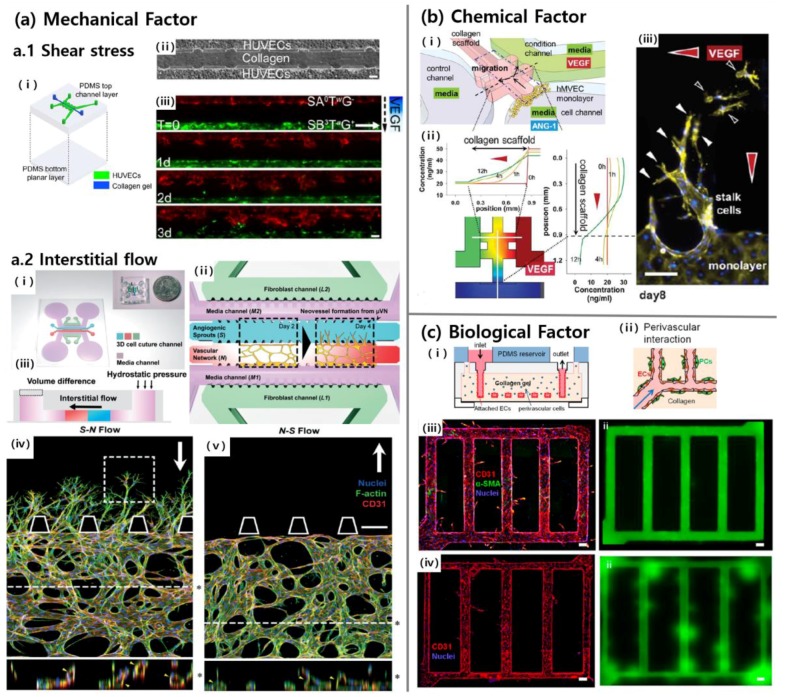
Three major factors (mechanical, chemical, and biological factors) in vessel formation on a chip. (**a.1**) Fluid flow was given by syringe pumps that were connected to reservoirs of the chip (i), which had one channel for collagen and two channels for human umbilical vein endothelial cells (HUVECs) (ii). Shear stress inhibited sprouting in the presence of interstitial flow and vascular endothelial growth factor (VEGF) gradient (iii) [12]. Scale bars 100 μm. (**a.2**) Angiogenic sprouting from the vascular network (i–ii) was observed in the presence of interstitial flow (iii). Sprouting was more active when the interstitial flow acts on sprouting in reverse direction (iv), than forward direction (v) [45]. Scale bar 200 μm. (**b**) The direction of sprouting could be guided along spatial VEGF gradient (iii) by forming two orthogonal gradient profiles on the gel region (i–ii) [21]. Scale bars 100 μm. (**c**) HUVECs were seeded on the hollow microchannels in the pericyte-embedded collagen gel (i) to generate stable vascular structures through heterotypic cell–cell interactions (ii). Pericytes (α-SMA, green) enclosed endothelial wall (CD31, red) and contributed to structural stability (iii) confirmed by perfusing of fluorescent microbeads (green). A number of leakages could be observed when only HUVECs were seeded (iv) [56]. Scale bars 100μm. Reproduced with permission.

The microfluidic device can control the mechanical factors through various ways. First, the syringe pump can be connected to the reservoir of the channel to provide a flow with a precise and constant flow rate (Figure 2a.1) [12]. It is also possible to create fluid flow through a hydrostatic pressure difference (Figure 2a.2) [45]. The size of the shear stress applied to the channel wall can also be controlled by varying the channel width, even though the channel is connected to a single syringe pump [26].

Fluid shear stress is confirmed as one of the dominant factors among mechanical factors [49,51,57]. The barrier function and stability related to the permeability of the vessel wall were examined for various values of flow rate, shear stress, transmural pressure, and average luminal pressure, and among different parameters, the fluid shear stress was determined as the most dominant factor [57]. Shear stress lowers the permeability and ultimately increases stability by narrowing vascular cells. Moreover, shear stress inhibits sprouting from the vascular wall through nitric oxide action, even in the presence of interstitial flow and vascular endothelial growth factor (VEGF) gradient (Figure 2a.1) [20].

Interstitial flow also plays a major role in vessel development. In particular, the sprouting behaviors are very different depending on the direction of interstitial flow acting upon the sprouting vessel (Figure 2a.2) [45]. Sprouting from existing vascular networks is most active in the reverse direction of interstitial flow. Additionally, given the gradient of the angiogenic factor with interstitial flow together, their influence on vasculatures may be greater. For instance, sprouting in both reverse interstitial flow and positive VEGF gradients showed a significant number of activated filopodial protrusions into the gel, but sprouting in both forward interstitial flow and negative VEGF gradients showed dilated morphologies [12].

Some attempts have been made to examine the effects of cellular mediators that respond to mechanical stimuli. For example, ECs regulate their morphology such as the polarization and alignment through the mechanism mediated by small GTPase RhoA in response to shear stress [58]. Furthermore, by culturing RhoA-blocked HUVECs in microfluidic devices, it has been confirmed that the RhoA-mediated mechanism plays a major role in VEGF-driven vascular development, especially extension, under shear flow [59]. Additionally, histone deacetylases have been identified to stimulate MMP14 expression in response to interstitial flow and VEGF [60].

### 3.2. Chemical Factors

Angiogenic growth factors are intensely involved in angiogenesis as the name suggests [1]. VEGF is the most important growth factor for blood vessel formation as it promotes EC migration and proliferation through VEGF receptor-2 signaling [61,62]. Most importantly, VEGF determines the direction of angiogenic sprouting by guiding the filopodial protrusion to a higher concentration of VEGF [2,63]. Other angiogenic growth factors such as platelet-derived growth factor (PDGF), transforming growth factor beta 1 (TGF-β1), and angiopoietin (Ang-1) enhance the stabilization of the vessel wall through different cellular mechanisms [2]. There have also been attempts to quantify the role of various angiogenic factors when they are selectively or simultaneously introduced [20,64].

The generation of a concentration gradient of angiogenic factors is crucial in vessel formation as it guides the directions of vasculogenesis or sprouting (Figure 2b). A linear profile of chemical concentrations can be formed in the hydrogel because the diffusion rate of a molecule is much lower in hydrogel than in the cell medium [65]. Therefore, various shapes of gradient profiles can be obtained by varying the configurations of microfluidic channels [9]. For example, a relatively simple device with a single gel channel between two media channels [66,67] or a device with two gel channels between three media channels can generate the linear gradient profiles across the gel channels [65]. In addition, a device with a gel scaffold enclosed by three channels can form two profiles of gradients in two orthogonal directions (Figure 2b) [21]. In these ways, different aspects of sprout formation can be examined under the gradients of each angiogenic factor.

### 3.3. Biological Factors

The examples of EC types commonly used for vascularization on chips are HUVECs, human microvascular ECs (HMVECs), and human artery ECs (HAECs). Although the differences in their functional characteristics regarding angiogenesis are poorly understood, there has been a study comparing the functional angiogenic ability of HAECs and HUVECs using microfluidic systems [68]. In the 3-D microenvironment, HAECs exhibited excellent angiogenic potential compared to HUVECs through particular mRNA upregulation, which was not revealed in a 2-D culture system. There have also been some attempts to increase physiological relevance by culturing organ-specific cell lines for mimicking the functionality of organs [22,69,70]. In addition, studies using hEPCs that have a substantial proliferative capacity were reported [42,71], and recently, specialized techniques for differentiating induced pluripotent stem cells (iPSCs) into ECs, which may enable personalized vasculature-on-a-chip, are being developed [71,72,73,74].

While it is difficult to consider in vivo neovasculature as functionally fully-developed vessels [75,76], the stability of in vivo neovasculature can be improved by co-culturing ECs with mural cells such as vascular smooth muscle cells, fibroblasts, and pericytes [77,78,79]. Structural robustness is improved as the mural cells gather and wrap around the neovasculature. Mural cells also induce paracrine signals by several signaling pathways, which greatly enhance the functional stability of the vascular wall [80,81,82].

A distinct advantage of microfluidic systems is the ability to co-culture multiple cell types on a single chip [56,83,84] as several types of cytokines are secreted by the cells during heterotypic cell interactions, and such paracrine signaling cannot be observed in a conventional single cell culture environment [85]. A number of attempts have been made to create mature and stable vascular networks by co-culturing ECs and mural cells in vitro (Figure 2c) [56,86]. Moreover, when BM-hMSCs and ECs are co-cultured in a microfluidic system, BM-hMSCs were differentiated into mural cells by interaction with ECs, and angiogenic sprouting was actively achieved [19,76,87,88,89]. Furthermore, it is possible to control cell–cell interactions spatiotemporally when co-culturing multiple cell types in a microfluidic device, by seeding cells in selective compartments or at different time points. Nonetheless, the functionality of engineered vasculature cannot last for more than a few weeks in current in vitro experimental setups [90,91,92]. We hope that creative methods will be developed to overcome this limitation.

## 4. Applications

Some applications have been developed to use a vasculature-on-a-chip to study diseases in vitro. These developments may allow the investigation of malfunctions of the vessel wall itself (e.g., endothelial dysfunction), as well as the involvement of blood vessels in the functional abnormality of the nearby tissue and organ (e.g., cancer). With the recent advancements in microfluidic technology, vast amounts of studies are undergoing, and we have listed the broad overview of applications in the table below (Table 1). Furthermore, in the following sections, we will introduce the particular examples of developed in vitro vascular disease models including vasculature-related organ regeneration on an in vitro chip and also describe drug screening platforms.

### 4.1. Endothelial Dysfunction

**Figure 3:**
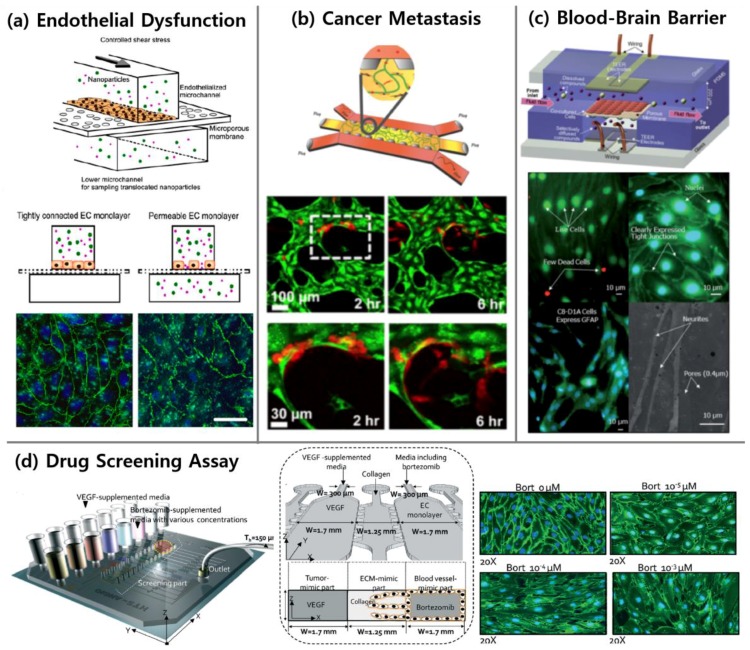
Representative figures of applications using vasculature-on-a-chip. (**a**) Increased permeability of inflamed endothelium was demonstrated with translocation of nanoparticles over an EC monolayer and immunostaining of VE-cadherin (green) and nuclei (blue) [98]. Scale bars 20 µm. (**b**) The bone microenvironment was replicated to investigate extravasation of breast cancer cells (red) from microvasculature (green) [23]. (**c**) Microfluidic model of the blood-brain barrier (µBBB) was characterized, and its validity was demonstrated [121]. (**d**) HUVECs morphology was investigated quantitatively depending on anti-angiogenic drug concentrations to find optimal effective concentration which minimizes both the number of migrated HUVECs and their morphological changes [24]. Reproduced with permission.

Endothelial dysfunction is a major physiological mechanism that can cause thrombosis, atherosclerosis, and inflammatory diseases [135,136,137]. Continuous efforts are being made to elucidate the underlying principles of endothelial dysfunction and related diseases, but with a considerable number of factors involved in such complex mechanisms, most studies using in vitro microfluidic models are somewhat limited in describing the phenomena [138,139,140,141]. Other examples of vasculature-on-a-chip confirmed that inflammatory endothelial activation mediated by tumor necrosis factor alpha (TNF-α) and disturbed flow [93,95] resulted in high permeable and prothrombotic states of the endothelium [56]. In particular, Kim et al., verified that increased endothelial permeability was due to inflammatory stimulation by probing nanoparticle translocation across an EC monolayer (Figure 3a) [98]. Other studies have demonstrated that atherosclerotic lesions or thrombus formation highly occurred at inflamed endothelial sites [25,94].

### 4.2. Vasculature-On-A-Chip and Cancer

Cancer is another area in which in vitro disease model research into the function of the vasculature is crucial. We have categorized and reviewed the vascular involvement in two following sections: tumor angiogenesis and cancer metastasis.

#### 4.2.1. Tumor Angiogenesis

During the generation of primary tumors, blood vessels affect nearby tumors by adjusting microenvironments that control several mechanical and chemical stimuli. They also provide oxygen and other nutrients to the tumor and play a major role in metastasis [142,143]. Therefore, a complete understanding of tumor angiogenesis is essential in treating the tumor [5]. Several studies utilized microfluidic devices to study tumor angiogenesis through co-culturing tumors and the endothelium. [33,144]. Particularly, Buchanan et al. investigated the correlation between shear stress and tumor-expressed angiogenic factors using a 3-D microfluidic tumor vascular model, where they co-cultured tumor and ECs [33]. EC migration was measured under different pH conditions of collagen gel and several combinations of cell types [144]. Different characteristics of tumor angiogenesis related to ECM conditions were also investigated [15,144].

#### 4.2.2. Cancer Metastasis

Cancer metastasis is of clinical importance because approximately 90% of cancer-related mortality is due to cancer metastasis [145]. It is recognized that extravasation of CTCs have exhibited great organ specificity [146], and it has also been confirmed that extravasation occurs more in specific microenvironments [147]. The multi-organ microfluidic chip designed by Xu et al., successfully reproduced growth, invasion, metastasis, and proliferation of lung cancer cells within the target organ [29]. Moreover, they confirmed that the extravasated lung cancer cells damaged distant organs through sensing the expressions of related proteins. Bersini et al. mimicked the process of breast cancer metastasis to bone by replicating the bone microenvironment with BM-hMSCs that secreted bone-marker proteins [10]. The higher metastasis rate was confirmed in this bone microenvironment condition. Furthermore, the generation of 3-D microvascular networks within the bone or muscle-mimicking microenvironments enabled the in vitro investigation of organ-specific cancer cell metastasis (Figure 3b) [23].

### 4.3. Vasculature in Organ-On-A-Chip

Blood vessels connect different organs in the body and contribute to the organ functions by providing nutrients and oxygen [148,149]. In this regard, numerous studies have been conducted to mimic physiologically appropriate responses by incorporating the vasculature into organs-on-chips (Table 1). In fact, we direct readers with special interests to other review articles that each describes a particular organ-on-a-chip model such as the lung, kidney, and liver [7,22,90,150]. Furthermore, we will focus this section on the reconstruction of the blood-brain barrier (BBB) and lymphatic system that are studied in in vitro microfluidic systems.

#### 4.3.1. Blood-Brain Barrier

The BBB is a physiological barrier of the central nervous system (CNS), which regulates the transport system between the brain and blood. The BBB disturbs the passage of drugs to the brain and restricts effective therapy for CNS diseases [151]. Additionally, the BBB can be a particular target because the malfunction of the BBB caused by neuronal diseases results in additional conditions [152]. Recently, the development of microfluidic devices mimicking the BBB in vitro has become a field of active research. Membrane-based multilayer devices are often used to investigate the BBB [121,123,124]. Booth et al. incorporated trans-endothelial electrical resistance electrodes in a device to demonstrate the validity of the in vitro BBB system (Figure 3c) [121]. Furthermore, co-culturing of ECs, pericytes, and glial cells improved the physiological relevance with the BBB [123]. There was also an attempt to recapitulate a neurovascular system in which neurons, astrocytes, and microglia formed the neural chamber and ECs constructed a vascular channel [124]. While most of the research on the BBB in microfluidics relied on membranes, some studies reported different techniques to circumvent the membrane usage. One study designed microholes in a microfluidic device and entrapped HUVECs [153]. The HUVECs trapped in the holes represented the engineered BBB, and the addition of an astrocyte-conditioned medium altered the permeability. Kim et al., fabricated a device with 3-D printing to mimic brain microvasculature [127]. In another example, HUVECs, astrocytes, and neurons were co-cultured within the collagen gel along the patterned microchannel for simulating physiological characteristics of the BBB [128].

#### 4.3.2. Lymphatic System

The lymphatic system is part of the circulatory system in the body, and its malfunction may attribute to various diseases such as inflammation and cancer metastasis [154,155]. Rare in vitro models have been reported to examine interactions between lymphatic endothelial cells (LECs) and their microenvironment for lymphangiogenesis [132,156,157]. In particular, Kim et al., quantified the angiogenic sprouting of LECs towards the fibrin matrix by controlling various growth factors and interstitial flow in microfluidic devices that contained fibroblasts-embedded in fibrin gel [130]. In addition, anti-lymphangiogenic drugs have also been tested to confirm the efficacy of common drug effects, demonstrating a well-designed in vitro lymphatic model.

### 4.4. Drug Screening Related to Vascular Diseases

Microfluidic systems offer new opportunities for pharmaceutical testing as the assays enable visualization of biological dynamics and reduction of the amount of cells and reagents required to evaluate their performance [24,158]. Dereli-Korkut et al., fabricated three-layered devices, and encapsulated cancer cells in hydrogel [159]. This microfluidic platform assessed the viability of cancer cells against anticancer compounds. Theberge et al., designed a cell-based assay by co-culturing HUVECs, fibroblasts, and macrophages [17]. They confirmed effects of the MMP12 inhibitor on vascularization and showed that cellular communications were critical to angiogenesis. The HUVEC monolayers generated by Kim et al., were used for quantitatively investigating the effects of an anti-angiogenic drug [24]. They monitored cell viability, gaps between ECs, and proliferation rates at different doses and multiple treatment times (Figure 3d). Bai et al., performed a study that treated injected cancer cell aggregates at different dosages of drugs to suppress the dissemination of cancer cells [160]. This study evaluated the singular and combined effects of drugs to obstruct the dissemination of cancer cells. Korin et al., mimicked vascular stenosis by incorporating a narrow channel and evaluated the efficacy of shear-targeting drugs [158]. The study estimating the performance of polymeric vehicles for gene delivery was also reported [161].

## 5. Conclusions and Future Perspectives

A microfluidic platform provides a systematic and controllable investigation of not only vascularization itself but also diseases that are closely linked to disorders of the vascular network. However, there are a few prerequisites for an epoch of microfluidics-based disease models. Firstly, further development of smooth integration methods for a flow system to vasculature-on-a-chip devices will be beneficial. While integration of the flow system is an essential part of providing a shear-driven biophysical stimulation to the in vitro system, maintaining constant flow requires a specific pump-integrated system or periodic filling of the medium in the case of the gravity-driven source. Secondly, the precision of the chemical gradient in a microfluidic device should be improved. Although chemical concentrations play a significant role in cell dynamics, even the latest methods are not capable of controlling the concentration gradient with the precision to fine tune cell dynamics. Another important future study could be investigating the effects of using different EC types as well as developing an in vitro lymphatic system. Most of the current efforts are placed using particular cell lines, but a proper demonstration of the characterization of available EC types including LECs will enhance the capacity for on-chip investigation of vascular diseases. Lastly, the durability of the engineered vessels in a microfluidic device should be improved. Due to several technical deficiencies, the engineered vasculature lasts for only a couple of weeks, limiting the current applications to acute illness and early stages of chronic diseases. Therefore, advances in a microfluidics-based disease model to overcome the limitations stated above will be a milestone for a new paradigm shift.

## Figures and Tables

**Table 1:** Summary of applications using engineered vasculature.

Applications	Objectives	Highlighted Features	References
Endothelial Dysfunction	Thrombosis	Stimulating thrombus formation by TNF-α	[56,93]
		Stimulating thrombus formation by mechanical cue	[93,94]
	Immune Response	Inflammatory endothelial activation	[95]
		Binding of T cells to ECs	[96]
		Neutrophil extravasation	[97]
	Atherosclerosis	Promoting thrombus formation under plaque geometry	[25,94]
		High permeability in atherosclerotic endothelium	[98]
Cancer	Tumor Angiogenesis	3-D tumor angiogenesis by controlling microenvironment	[15,33,99,100,101,102,103,104,105,106]
	Cancer Metastasis	(Organ-specific) Extravasation	[23,107,108,109,110]
		Intravasation	[39,110]
		Adhesion of CTCs to endothelium	[111]
Organ Regeneration	Lung	Engineering functional alveolar-capillary interface	[112,113]
	Heart	Engineering functional cardiac tissue	[114,115]
	Liver	Engineering functional hepatic tissue	[69,115,116]
	Kidney	Engineering functional renal tissue	[117,118]
	Artery	Mimicking 3-D artery architecture	[119]
	Skin	Co-culturing of skin equivalents with vascular cells	[120]
	Blood	Membrane-based cell culture	[121,122,123,124,125]
	-Brain	Gel-based cell culture	[126,127,128]
	Barrier	Validating functionality by TEER measurement	[121,122,123,129]
	Lymphatic System	Lymphangiogenesis	[130,131,132]
Drug Screening		Identifying effects of drug	[113,133,134]
